# Different tropism of adenoviruses and adeno-associated viruses to corneal cells: implications for corneal gene therapy

**Published:** 2008-11-18

**Authors:** J. Liu, M. Saghizadeh, S.S. Tuli, A.A. Kramerov, A.S. Lewin, D.C. Bloom, W.W. Hauswirth, M.G. Castro, G.S. Schultz, A.V. Ljubimov

**Affiliations:** 1 Department of Molecular Genetics and Microbiology, University of Florida, Gainesville, FL; 2 Department of Ophthalmology, University of Florida, Gainesville, FL; 3 Department of Obstetrics and Gynecology, University of Florida, Gainesville, FL; 4 Ophthalmology Research Laboratories, Cedars-Sinai Medical Center, Los Angeles, CA; 5 Gene Therapeutics Research Institute, Cedars-Sinai Medical Center, Los Angeles, CA; 6 David Geffen School of Medicine at UCLA, Los Angeles, CA

## Abstract

**Purpose:**

Diseased corneas are potential targets for viral-based gene therapy to normalize (stimulate or inhibit) the expression of specific proteins. The choice of viral vectors is important to achieve optimal effect. The purpose of this study was to compare the tropism to different corneal cells of recombinant adenovirus (rAV) and recombinant adeno-associated virus (rAAV) constructs using live rabbit and organ-cultured human corneas.

**Methods:**

rAV constructs harbored the green fluorescent protein (*GFP*) gene under the control of major immediate early cytomegalovirus (CMV) promoter. rAAV constructs from virus serotypes 1, 2 5, 7, and 8 had *GFP* under the chicken β-actin promoter and CMV enhancer. For organ culture, 16 healthy and diabetic postmortem human corneas were used. Five or fifteen μl rAV at 10^7^ plaque forming units per 1 μl were added for 2 days to culture medium of uninjured corneas that were further cultured for 5–32 days. rAAV were added at 1.2–7.8×10^10^ vector genomes per cornea for 3 days to each cornea; the culture then continued for another 14–23 days. Corneal cryostat sections were examined by immunohistochemistry. Live rabbit corneas were used following excimer laser ablation of the corneal epithelium with preservation of the basal cell layer. Equal numbers of rAAV particles (2x10^11^ vector genomes) were applied to the cornea for 10 min. After seven days to allow for corneal healing and gene expression the animals were euthanized, the corneas were excised, and sections analyzed by immunohistochemistry.

**Results:**

By direct fluorescence microscopy of live organ-cultured human corneas GFP signal after rAV transduction was strong in the epithelium with dose-dependent intensity. On corneal sections, GFP was seen in all epithelial layers and some endothelial cells but most keratocytes were negative. In rAAV-transduced organ-cultured human corneas GFP signal could only be detected with anti-GFP antibody immunohistochemistry. GFP was observed in the epithelium, keratocytes, and endothelium, with more pronounced basal epithelial cell staining with rAAV1 than with other serotypes. No difference in the GFP expression patterns or levels between normal and diabetic corneas was noted. The rabbit corneas showed very similar patterns of GFP distribution to human corneas. With all rAAV serotype vectors, GFP staining in the epithelium was significantly (p=0.007) higher than the background staining in non-transduced corneas, with a trend for rAAV1 and rAAV8 to produce higher staining intensities than for rAAV2, rAAV5 (p=0.03; rAAV5 versus rAAV1), and rAAV7. rAAV serotype vectors also transduced stromal and endothelial cells in rabbit corneas to a different extent.

**Conclusions:**

rAAV appears to reach many more corneal cells than rAV, especially keratocytes, although GFP expression levels were lower compared to rAV. rAV may be more useful than rAAV for gene therapy applications requiring high protein expression levels, but rAAV may be superior for keratocyte targeting.

## Introduction

Inherited, iatrogenic, and metabolic corneal diseases are commonly encountered and present serious clinical problems, often calling for corneal transplantation. Some of them have a known underlying gene defect, best exemplified by a group of dystrophies caused by a mutation in transforming growth factor-β-induced gene, *TGFBI/BIGH3* [1-4]. Other diseases, like keratoconus, also have a genetic component, but the altered gene(s) still remains unknown [5,6]. Many of these disorders could be potentially treated by supplying a functional gene or changing the expression levels of specific genes in specific cells [7,8]. Another potential area of corneal gene therapy application is treatment of fibrotic disorders and of corneal haze after refractive procedures [7,9,10]. Our data suggested the importance of altered expression of proteinases and growth factors in diabetic corneas [11-13], which could also be potentially corrected by gene therapy to alleviate the symptoms of diabetic keratopathy.

In recent years, viral and non-viral gene therapy has been successfully attempted for delivering genes into corneal epithelial, stromal, and endothelial cells [14-20]. Although most studies used reporter genes, such as green fluorescent protein (GFP), some groups showed improved corneal parameters after alkali burns or reduction of pathological neovascularization after introduction of specific genes of interest [19,21].

The advantage of viral-based therapies is that viruses easily enter the cells, are more efficient than other gene delivery vehicles, and enable good expression of secreted proteins [22-24]. Recombinant adenovirus (rAV), adeno-associated virus (rAAV), herpes simplex virus type 1, and lentiviruses are commonly used viral vectors. It has been emphasized that some viral vector treatments induce unacceptable immune responses, inflammation, and virus integration into the host genome, with unpredictable consequences [25,26]. However, recently introduced new-generation recombinant viruses combine long-term effects, high gene transfer efficiency, and the ability to transfect non-dividing cells with lack of a significant immune response. They are rapidly becoming vehicles of choice for gene therapy. The on-off mechanisms also allow time-controlled action of viral vectors [27]. Viral vectors have been used to deliver specific genes with high efficiency to eye tissues including cornea [14,19,28,29].

Recent studies of rAAV-driven gene transduction found that the preferential tissue site for expression and its relative levels were dependent on virus serotype and route of administration [30-32]. It seems, therefore, important to optimize viral vectors for each tissue of interest in terms of cell tropism and expression levels. The main goal of the present work was to compare the corneal cell tropism and levels of reporter protein GFP expression driven by different strains of rAAV or rAV. In situ localization of transgene expression in corneal tissue might provide guidelines for selecting the correct viral vector to transduce the cell type of interest. Two different systems were used for rAAV transduction, rabbit eyes and human corneal organ cultures. For rAV, only human corneal organ cultures were used because this vector has been previously evaluated in the rat eye model in the same way as has been done here in the rabbit eyes [33]. The presented results show that rAV ex vivo mostly infects epithelial and also endothelial cells, whereas rAAV can infect most or all corneal cells both in vivo and ex vivo.

## Methods

### Production of rAAV serotype vectors

Recombinant AAV pseudo-serotypes 1, 2, 5, 7, and 8 were constructed by using ITR and Rep from serotype 2 AAV and capsid protein from serotypes 1, 2, 5, 7, and 8, respectively [34-37]. All rAAV vectors (serotypes 1, 2, 5, 7, and 8, and true type 2) harbor the *GFP* gene driven by chicken β-actin promoter and cytomegalovirus (CMV) enhancer. AAV purification was performed as previously described [38,39].

### Production of rAV vectors

The coding region of *GFP* was cloned into the unique BamHI restriction site of the transfer vector pAL119 under the control of immediate early CMV promoter and upstream of a polyadenylation signal on a HindIII flanked expression cassette. The orientation of the cloned transgene was determined by restriction analysis [40]. rAV was generated by homologous recombination in HEK-293 cells following co-transfection of the *GFP*-harboring shuttle vector pAL119 with the plasmid pJM17 (Microbix Biosystems, Toronto, Canada) by calcium phosphate co-precipitation [40,41]. Homologous recombination resulted in the generation of the replication deficient rAV vector. rAV was propagated in HEK-293 cells, purified twice on CsCl_2_ gradients, and dialyzed twice against 10 mM Tris-HCl pH 7.5, 1 mM MgCl_2_, 135 mM NaCl, and once against the same buffer with 10% glycerol. The virus was titrated by plaque assay on HEK-293 cells. Usually, viral titers are 10^11^-10^12^ plaque-forming units per ml (pfu/ml). GFP staining appears to be dependent on virus dose, with most intense staining at doses of 10^8^ to 10^9^ pfu/ml.

### Rabbit eye model

The delivery efficiency of a transgene packaged in different rAAV serotypes was tested in live rabbit corneas. Adult New Zealand white rabbits were used for this study and the procedure was performed in accordance to the animal care guidelines published by the Institute for Laboratory Animal Research (Guide for the Care and Use of Laboratory Animals). Briefly, rabbits were anesthetized with isofluorane/oxygen inhalation, proparacaine eye drops were applied to achieve local anesthesia of corneas, and both eyes of each rabbit were ablated to a depth of 25 μm with a VISX 20/20 excimer laser, creating a 6.5 mm diameter central epithelial injury. The ablation conditions were specifically designed not to remove all of the corneal epithelial cell layers, but to only ablate to the basal cell layer. This procedure was exactly like the one described previously for rat eyes treated with rAV [33]. The intention was to transduce the basal epithelial cells and the stromal fibroblasts by topical application of rAAV vectors. The ablated areas of both eyes of each rabbit were then exposed for 10 min to 2×10^11^ rAAV vector genomes applied on the cornea in phosphate buffered saline in a 10 mm diameter vacuum trephine, which contained the rAAV vector. Overall, two rabbit corneas were treated with each of the rAAV vector serotypes.

Seven days after excimer laser ablation and transduction by rAAV vectors, the animals were euthanized, and the corneas were briefly fixed in situ by flooding the eye with 4% *p-*formaldehyde. Corneas were then excised, fixed overnight, embedded in Tissue Tek OCT Compound (Sakura Finetek, Torrance, CA) and frozen by dipping into liquid nitrogen-cooled isopentane. Tissue sectioning was performed with a Microm H550 cryostat (Microm, Walldorf, Germany) and 10 μm sections were mounted on Superfrost/Plus microscope slides (Fisher Scientific, Pittsburgh, PA) for immunohistochemistry. Indirect immunostaining was performed using chicken anti-GFP (Millipore, Temecula, CA) followed by a biotinylated secondary antibody (Promega, Madison, WI). Two types of signal detection systems were used. Streptavidin-peroxidase (Vector Laboratories, Burlingame, CA) was used for light microscopy with NovaRed substrate (Vector Laboratories). Alkaline phosphatase detection system with VectorRed substrate (Vector Laboratories) was used in the fluorescent regime with a Leica TCS SP2 AOBS Spectral Confocal Microscope (Leica Microsystems, Bannockburn, IL).

**Figure 1:**
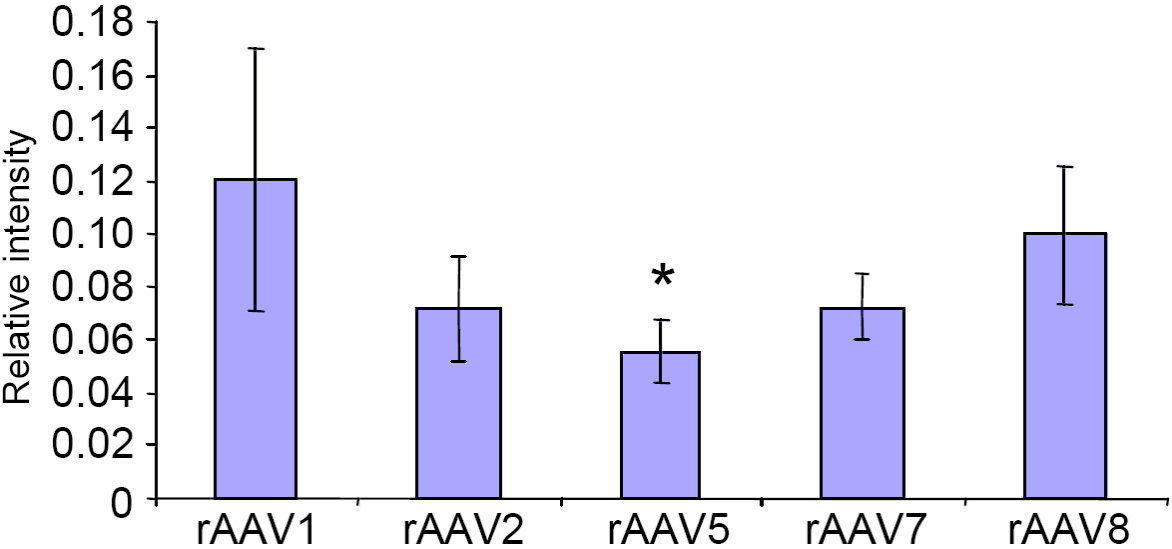
Quantitation of the GFP intensity of staining in corneal epithelium. The strongest signal is observed with rAAV1 and rAAV8, with other serotypes being weaker. The asterisk indicates a p=0.03 (rAAV5 versus rAAV1).

For the quantification of GFP expression from different serotypes of rAAV in rabbit cornea, bright-field micrographs were taken for each section using a morphometric microscope, with the same setting at the same day at a magnification of 20X, and the relative level of GFP immunostaining was measured using MCID software (InterFocus Imaging, Linton, UK). Basically, sampling tools of MCID were used to trace the epithelial region of each image, and the semiquantitative densitometry function was used to evaluate the opacity of the selected region. After averaging the score from images of each treatment, the score for background (control treatment) was subtracted to generate the chart in Figure 1. Levels of immunostaining determined by the MCID software were analyzed for statistical significance by ANOVA followed by the LSD post-hoc test using Statistica™ software (StatSoft^®^, Tulsa, OK).

### Corneal organ culture

Normal and diabetic postmortem organ-cultured human corneas (a total of 16) were used. These were obtained from National Disease Research Interchange (NDRI, Philadelphia, PA). NDRI has a managerial committee that operates under NIH oversight. It ensures adequate donor family consent and patient information protection, as well as compliance with human subject regulations. Both normal and diabetic corneas were included because potential viral gene therapy for diabetic keratopathy necessitates comparative testing of different transduction vectors to optimize the conditions of gene delivery. Cultures were initiated and maintained as described previously, in serum-free medium at the air-liquid interface [42]. After 2–3 days in culture, viral constructs harboring *GFP* were added to the corneas. Because rAV titer may influence reporter gene expression [43], two titers were used (0.5×10^8^ and 1.5×10^8^ pfu per cornea); however, they produced similar results (data not shown). Two non-diabetic corneas each from two different donors received the lower dose, and another two, the higher one. Virus was incubated for 2 days in culture medium with uninjured corneas, which were further cultured for either 5 days (for efficiency assessment) or 32 days (for duration of detectable expression). rAAV constructs from virus serotypes 1, 2, and 8 were used and were the same as in the rabbit model. For each serotype, four organ-cultured corneas were used from four different donors including two non-diabetics and two diabetics. rAAV vectors were added to corneas for 3 days at 7.8×10^10^ vector genomes per cornea for rAAV1, 3.5×10^10^ for rAAV2, and 1.2×10^10^ or 2.4×10^10^ for rAAV8 (in the range published previously [35]). The medium was then changed and the culture was continued for another 14 to 23 days, to assess transduction efficiency of corneal cells at different time points within the reported range of detectable GFP expression [7,33]. This was important to determine a potential therapeutic window for further clinical application. Live corneas transduced by rAV were visualized under a fluorescent microscope and periodically photographed; following rAAV transduction, it was not possible to detect fluorescent signal from live corneas. At the end of the experiment corneas were cut in half and embedded in OCT without fixation. For rAV, cryostat sections were examined by direct fluorescence microscopy. For rAAV, sections were fixed in 2% formalin for 5 min and subjected to indirect immunofluorescent staining with two different antibodies to GFP (rabbit ab6662; Abcam, Cambridge, MA, and mouse MAB3580; Millipore, Billerica, MA). Secondary cross-species adsorbed antibodies were from Jackson ImmunoResearch Laboratories (West Grove, PA). To assess the specificity of GFP antibodies, sections of organ-cultured corneas not exposed to GFP expressing vectors were stained.

## Results

### Rabbit eyes

**Figure 2:**
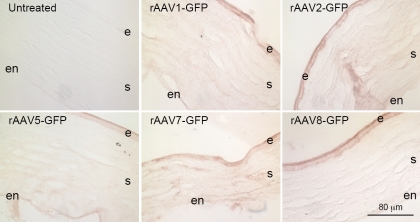
Immunostaining of rabbit corneas for GFP expression delivered by different serotypes of rAAV vectors, using the streptavivin-peroxidase detection system. e, epithelium; s, stroma; en, endothelium. Bar=80 μm.

Transduction with all rAAV serotypes (type 1, 2, 5, 7, and 8) led to GFP expressions in the rabbit cornea at seven days post-inoculation as indicated by immunostaining using the anti-GFP antibody. Representative images of GFP staining from rabbits’ eyes treated with different AAV serotypes as well as control eyes are shown in Figure 2. Substantial staining was observed in all cell types including epithelium, keratocytes and endothelium. The expression of GFP was not limited to the cells in the area covered by the trephine. No substantial difference was noticed in the GFP expression level along the corneal sections that included the limbal region. The wounded area could not be detected in the sections; therefore, the “renewed” or healed epithelium could not be distinguished from the epithelium in the noninjured area. However, transgene expression was detected throughout the corneal epithelial cell layers when the injury had healed. These results suggest that the rAAV vectors transduced both cells that “healed” the excimer laser ablation wound, such as the basal epithelial cells, and the cells that were outside of the wound area.

All rAAV serotypes transduced the corneal epithelial cell layer and produced GFP expression that was significantly (p=0.007) above levels in non-transduced corneas (background). Relative GFP immunostaining intensities in corneal epithelium from different rAAV serotypes are presented in Figure 1. Statistical analysis showed that rAAV1 yielded significantly higher GFP expression than rAAV5 (2.2 fold; p=0.03), with a trend (p=0.09) for higher expression than rAAV7, but was not significantly different from rAAV2 or rAAV8. There was also a trend for rAAV8 GFP expression to be higher than rAAV5 GFP expression (p=0.09).

**Figure 3:**
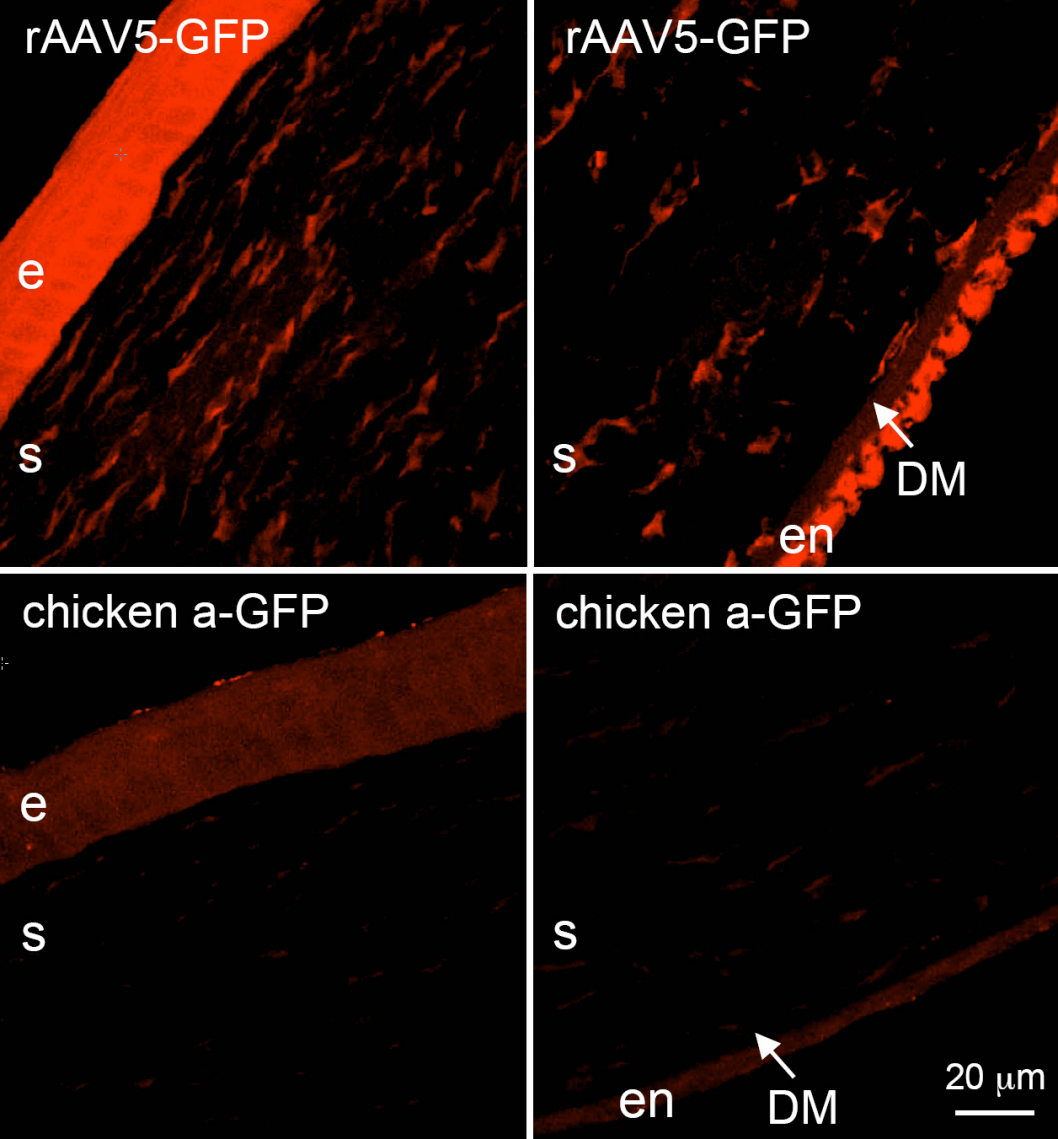
rAAV-GFP transduction of rabbit corneas. Upper row, rAAV-GFP5; strong signal is seen in the epithelium (e), stromal cells (s), and endothelium (en). Epithelial, stromal, and endothelial cells are positive for GFP after rAAV treatment. Lower row, untreated rabbit corneas as a negative control showing background fluorescence. DM indicates Descemet’s membrane. GFP was visualized using confocal microscopy and alkaline phosphatase fluorescent detection system. Bar=20 μm.

GFP expression in the stromal and endothelial layers of corneas transduced by all five rAAV serotypes was less than that observed in the epithelial cell layers. To increase sensitivity of detection, confocal microscopy was performed on immunostained sections using alkaline phosphatase-conjugated secondary antibody with the fluorescent VectorRed^®^ substrate. As shown in Figure 3, transduction with rAAV5 generated distinct immunostaining of stromal keratocytes and endothelial cells compared to non-transduced corneas. In contrast, transduction with rAAV1 and especially rAAV8 produced substantially weaker immunostaining of keratocytes and endothelial cells (Figure 2).

### Organ culture

**Figure 4:**
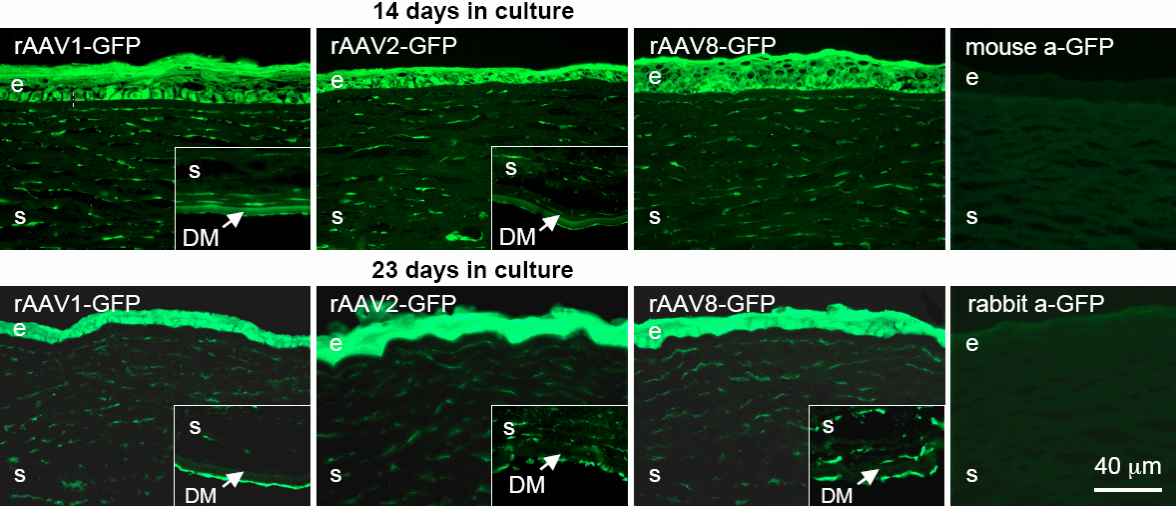
rAAV-GFP transduction of organ-cultured human normal and diabetic corneas. Virus serotypes 1, 2, and 8 were used. Upper row – staining with mouse a-GFP MAB3580 (right panel – staining of an organ-cultured cornea not exposed to rAAV-GFP [negative control] with the same antibody [mouse a-GFP]); lower row – staining with rabbit a-GFP antibody ab6662 (right panel – staining of another negative control cornea with antibody ab6662 [rabbit a-GFP]). Epithelial (e), stromal (s), and endothelial (inset panels) cells are well transduced by rAAV-GFP. No staining is seen in corneas not exposed to the GFP-expressing virus. Both antibodies give very similar patterns. GFP was revealed by indirect immunofluorescent staining. DM, Descemet’s membrane. Bar=40 μm.

GFP expression was also studied in rAAV-infected organ-cultured human normal and diabetic corneas. As in rabbit eyes, the signal intensity was low at both 14 and 23 days and could not be reliably detected with direct fluorescence microscopy. Immunohistochemistry with anti-GFP antibodies, however, allowed easy detection of the GFP signal (Figure 4). Epithelial cells were uniformly stained; only with rAAV1 the staining of basal cells was more pronounced than of other epithelial layers. At over three weeks, epithelial staining was somewhat stronger than at two weeks (Figure 4, compare top and bottom). In addition, positive staining of most or all keratocytes and endothelial cells was evident with all serotypes, in accordance with rabbit corneas (Figure 4). Normal and diabetic corneas showed no difference in GFP intensity and distribution (data not shown).

**Figure 5:**
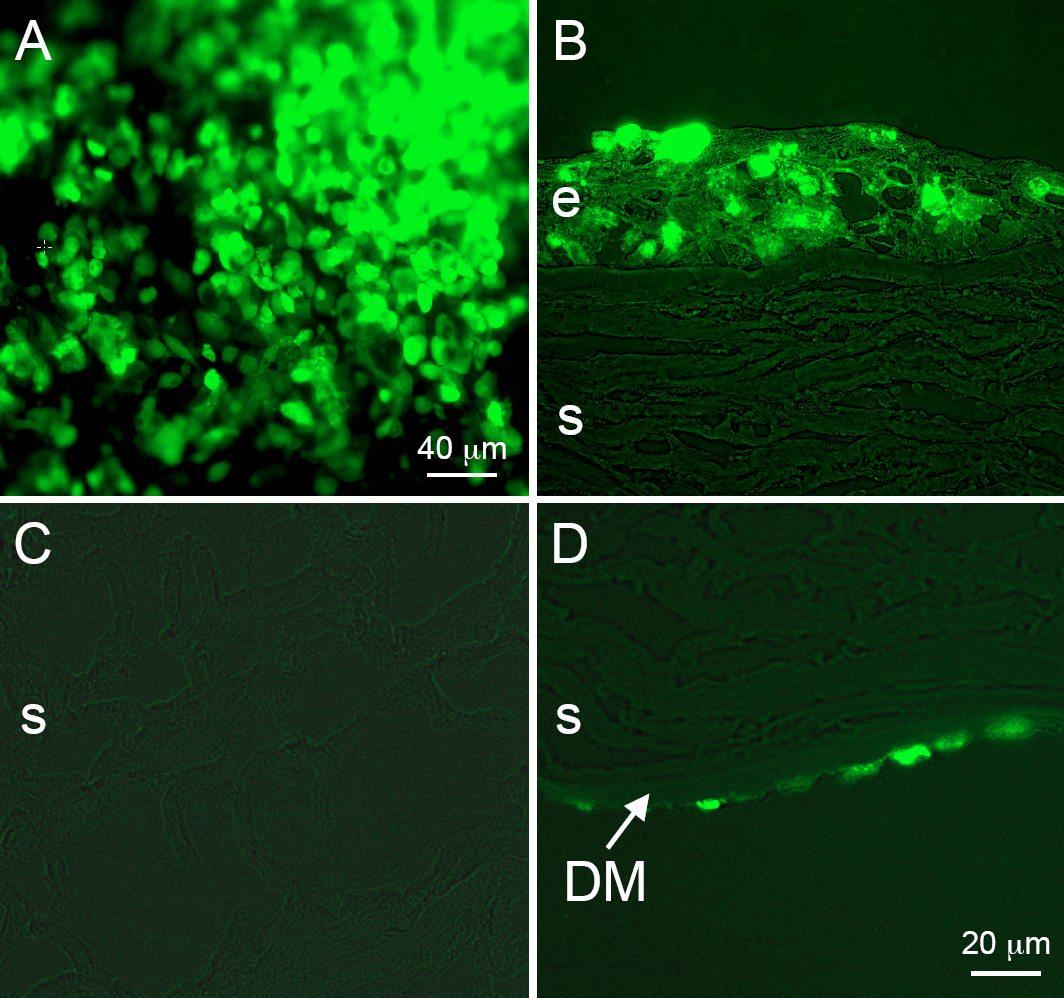
rAV-GFP transduction of organ-cultured normal corneas. Upper left, live cornea; other panels, transverse corneal sections (combined fluorescence and transmitted light). Mostly epithelial (**A**,**B**) and some endothelial cells (**D**) are transgene-positive, whereas stromal keratocytes (**C**) are negative. e, epithelium; s, stroma; DM, Descemet’s membrane. Direct fluorescence. Bar=20 μm.

Organ-cultured corneas were additionally used to study GFP expression after infection with rAV-GFP. Direct fluorescence microscopy of live corneas five days after rAV-GFP treatment revealed strong signal in the epithelium (Figure 5, upper left). The signal intensity correlated with the dose of the virus (data not shown). On corneal sections, GFP was clearly expressed in all epithelial layers; some negative cells were also seen (Figure 5, upper right). Most keratocytes were negative but some endothelial cells were positive (Figure 5, lower row). The expression of GFP after rAV-GFP infection was observed for more than one month and started declining thereafter, consistent with previous data [44].

## Discussion

Adenoviral, adeno-associated, and lentiviral vectors have been studied extensively for gene therapy. Currently, their widespread use is hampered by a relative lack of specific cell targeting, development of immune reactions (rAV, rAAV), and random integration into the host genome (lentiviruses) sometimes resulting in serious side effects [45,46]. However, corneal immune privilege makes it a potentially safe and promising site for viral gene therapy. This is particularly relevant for rAV [47]. In case of rAAV, there is an added advantage that the virus is non-pathogenic and is able to more readily infect not only dividing but also non-dividing cells. An important question in viral gene therapy remains the vector tropism for different cells and a possibility of infecting only their specific subsets.

The data presented here suggest that both rAV and rAAV may be used to successfully deliver genes of interest into human corneas. The rAAV data on organ-cultured human corneas including GFP distribution patterns and the necessity of using immunostaining to detect signal are very similar to our results obtained with the in vivo rabbit corneas (compare Figure 3 with Figure 4). rAAV may have some advantage over rAV in terms of longer persistence in vivo of delivered gene’s expression [48]. Moreover, as shown here, it was able to deliver the reporter gene into epithelial cells, keratocytes, and endothelium. The extensive transduction of all three layers of the cornea is likely to be due to the use of the vacuum trephine. Compared to previous studies, it ensured longer exposure of the rAAV vectors to the corneal cells at the ablation site. We have had only minimal success in transducing rabbit corneal cells after ablation unless the contact time on the corneal surface was prolonged through the use of the vacuum trephine. Just putting eye drops containing a rAAV vector on the rabbit cornea after ablation does not produce high levels of transduction of corneal cells.

Transduction of the rabbit corneas with rAAV8 and especially, rAAV1, produced a stronger GFP expression in epithelial cells compared to rAAV5. The stronger transgene expression with rAAV1 compared to rAAV5 was similar to previous data on transduction of erythropoietin gene into the retina with subretinal injection, and on transduction of apolipoprotein A-I_Milano_ with intramuscular or intravenous injection [31,49]. At the same time, rAAV1 yielded relatively less GFP expression in stromal keratocytes and endothelial cells than rAAV2 and rAAV5. These results could be due to different tropism of the rAAV serotypes for the three corneal cell types, similar to the situation in retina [31]. Serotype differences in stromal and endothelial transduction were not obvious in organ cultures, which could be due to easier accessibility of these cells to the virus. Our results agree with previous data showing that rAAV serotypes 1–5 ensured equal levels of transduction in cultured cells, but in vivo (liver and muscle) there were significant differences among serotypes in terms of gene expression levels [35].

The delivery method of rAAV vector in our in vivo study does not rely on injection into the cornea, which is painful and can cause damage to corneal structure. Our positive results with rAAV infection of corneal organ cultures suggest that in vivo gene transfer via rAAV may not even necessitate prior epithelial removal. Further studies need to be conducted to compare the penetration ability of each serotype of rAAV and duration of gene expression in vivo. The small sample number (two eyes of one rabbit) in each treatment may limit the representation of the observation, although it was consistent between serotypes. Future experiments to observe rAAV transgene expression at a longer period will provide more information. In addition, since self-complementary rAAV has been shown to lead to earlier expression of passenger genes, it might be useful to test this vector in the cornea.

rAV infection resulted mostly in gene transfer to the corneal epithelium, with less signal seen in corneal endothelial cells. Generally, there was no signal in keratocytes. The immunostaining was corroborated by quantitative RT–PCR after infection of organ-cultured corneas with rAV harboring c-met gene. Its expression was upregulated in isolated epithelial cells 2.3 fold on average (n=3) but stayed the same in the stromal cells (data not shown). GFP expression level was significantly higher with rAV than with rAAV. In agreement with our results, investigators observed that GFP-positive cells had a restricted distribution in the stroma following intrastromal injection of rAV, and there was no transfection of the epithelium or endothelium [50]. Our results differ from the in vivo rat model [33] in that in organ culture, more epithelial cells were transduced and endothelial cells were also positive. This is probably due to a much longer exposure to the virus in vitro and accessibility of the endothelial cells to rAV in the organ culture system.

Depending on whether the expression level or the ability to reach more corneal cells is more important, rAV or rAAV may be chosen for specific purposes of gene transfer to corneas. For instance, keratoconus is significantly associated with stromal changes [51,52], and potential gene therapy may use rAAV to target keratocytes. In case of diabetic corneas, where most abnormalities are associated with the epithelium [53], or if there is need for high levels of transgene expression, rAV may become a vector of choice as a gene therapy vehicle.

## References

[1] Vincent AL, Patel DV, McGhee CN (2005). Inherited corneal disease: the evolving molecular, genetic and imaging revolution.. Clin Experiment Ophthalmol.

[2] Kannabiran C, Klintworth GK (2006). TGFBI gene mutations in corneal dystrophies.. Hum Mutat.

[3] Pieramici SF, Afshari NA (2006). Genetics of corneal dystrophies: the evolving landscape.. Curr Opin Ophthalmol.

[4] Poulaki V, Colby K (2008). Genetics of anterior and stromal corneal dystrophies.. Semin Ophthalmol.

[5] Rabinowitz YS (2003). The genetics of keratoconus.. Ophthalmol Clin North Am.

[6] Aldave AJ, Bourla N, Yellore VS, Rayner SA, Khan MA, Salem AK, Sonmez B (2007). Keratoconus is not associated with mutations in COL8A1 and COL8A2.. Cornea.

[7] Mohan RR, Sharma A, Netto MV, Sinha S, Wilson SE (2005). Gene therapy in the cornea.. Prog Retin Eye Res.

[8] Kao WW (2006). Ocular surface tissue morphogenesis in normal and disease states revealed by genetically modified mice.. Cornea.

[9] Behrens A, Gordon EM, Li L, Liu PX, Chen Z, Peng H, La Bree L, Anderson WF, Hall FL, McDonnell PJ (2002). Retroviral gene therapy vectors for prevention of excimer laser-induced corneal haze.. Invest Ophthalmol Vis Sci.

[10] Saika S, Yamanaka O, Sumioka T, Miyamoto T, Miyazaki K, Okada Y, Kitano A, Shirai K, Tanaka S, Ikeda K (2008). Fibrotic disorders in the eye: Targets of gene therapy.. Prog Retin Eye Res.

[11] Saghizadeh M, Brown DJ, Castellon R, Chwa M, Huang GH, Ljubimova JY, Rosenberg S, Spirin KS, Stolitenko RB, Adachi W, Kinoshita S, Murphy G, Windsor LJ, Kenney MC, Ljubimov AV (2001). Overexpression of matrix metalloproteinase-10 and matrix metalloproteinase-3 in human diabetic corneas: a possible mechanism of basement membrane and integrin alterations.. Am J Pathol.

[12] Saghizadeh M, Chwa M, Aoki A, Lin B, Pirouzmanesh A, Brown DJ, Ljubimov AV, Kenney MC (2001). Altered expression of growth factors and cytokines in keratoconus, bullous keratopathy and diabetic human corneas.. Exp Eye Res.

[13] Saghizadeh M, Kramerov AA, Tajbakhsh J, Aoki AM, Wang C, Chai NN, Ljubimova JY, Sasaki T, Sosne G, Carlson MR, Nelson SF, Ljubimov AV (2005). Proteinase and growth factor alterations revealed by gene microarray analysis of human diabetic corneas.. Invest Ophthalmol Vis Sci.

[14] Mohan RR, Schultz GS, Hong JW, Mohan RR, Wilson SE (2003). Gene transfer into rabbit keratocytes using AAV and lipid-mediated plasmid DNA vectors with a lamellar flap for stromal access.. Exp Eye Res.

[15] Jessup CF, Brereton HM, Coster DJ, Williams KA (2005). In vitro adenovirus mediated gene transfer to the human cornea.. Br J Ophthalmol.

[16] Lai L, Lin K, Foulks G, Ma L, Xiao X, Chen K (2005). Highly efficient ex vivo gene delivery into human corneal endothelial cells by recombinant adeno-associated virus.. Curr Eye Res.

[17] Chen Z, Mok H, Pflugfelder SC, Li DQ, Barry MA (2006). Improved transduction of human corneal epithelial progenitor cells with cell-targeting adenoviral vectors.. Exp Eye Res.

[18] Sonoda S, Tachibana K, Uchino E, Okubo A, Yamamoto M, Sakoda K, Hisatomi T, Sonoda KH, Negishi Y, Izumi Y, Takao S, Sakamoto T (2006). Gene transfer to corneal epithelium and keratocytes mediated by ultrasound with microbubbles.. Invest Ophthalmol Vis Sci.

[19] Klausner EA, Peer D, Chapman RL, Multack RF, Andurkar SV (2007). Corneal gene therapy.. J Control Release.

[20] Parker DG, Kaufmann C, Brereton HM, Anson DS, Francis-Staite L, Jessup CF, Marshall K, Tan C, Koldej R, Coster DJ, Williams KA (2007). Lentivirus-mediated gene transfer to the rat, ovine and human cornea.. Gene Ther.

[21] Saika S, Yamanaka O, Okada Y, Miyamoto T, Kitano A, Flanders KC, Ohnishi Y, Nakajima Y, Kao WW, Ikeda K (2007). Effect of overexpression of PPARgamma on the healing process of corneal alkali burn in mice.. Am J Physiol Cell Physiol.

[22] Klebe S, Sykes PJ, Coster DJ, Bloom DC, Williams KA (2001). Gene transfer to ovine corneal endothelium.. Clin Experiment Ophthalmol.

[23] Yoo J, Choi S, Hwang KS, Cho WK, Jung CR, Kwon ST, Im DS (2006). Adeno-associated virus-mediated gene transfer of a secreted form of TRAIL inhibits tumor growth and occurrence in an experimental tumor model.. J Gene Med.

[24] Wei K, Kuhnert F, Kuo CJ (2008). Recombinant adenovirus as a methodology for exploration of physiologic functions of growth factor pathways.. J Mol Med.

[25] Lowenstein PR, Mandel RJ, Xiong WD, Kroeger K, Castro MG (2007). Immune responses to adenovirus and adeno-associated vectors used for gene therapy of brain diseases: the role of immunological synapses in understanding the cell biology of neuroimmune interactions.. Curr Gene Ther.

[26] Li Q, Miller R, Han PY, Pang J, Dinculescu A, Chiodo V, Hauswirth WW (2008). Intraocular route of AAV2 vector administration defines humoral immune response and therapeutic potential.. Mol Vis.

[27] Curtin JF, Candolfi M, Xiong W, Lowenstein PR, Castro MG (2008). Turning the gene tap off; implications of regulating gene expression for cancer therapeutics.. Mol Cancer Ther.

[28] Jun AS, Larkin DF (2003). Prospects for gene therapy in corneal disease.. Eye.

[29] Rosenblatt MI, Azar DT (2004). Gene therapy of the corneal epithelium.. Int Ophthalmol Clin.

[30] Mochizuki S, Mizukami H, Kume A, Muramatsu S, Takeuchi K, Matsushita T, Okada T, Kobayashi E, Hoshika A, Ozawa K (2004). Adeno-associated virus (AAV) vector-mediated liver- and muscle-directed transgene expression using various kinds of promoters and serotypes.. Gene Ther Mol Biol.

[31] Lebherz C, Maguire A, Tang W, Bennett J, Wilson JM (2008). Novel AAV serotypes for improved ocular gene transfer.. J Gene Med.

[32] Zincarelli C, Soltys S, Rengo G, Rabinowitz JE (2008). Analysis of AAV serotypes 1–9 mediated gene expression and tropism in mice after systemic injection.. Mol Ther.

[33] Igarashi T, Miyake K, Suzuki N, Kato K, Takahashi H, Ohara K, Shimada T (2002). New strategy for *in vivo* transgene expression in corneal epithelial progenitor cells.. Curr Eye Res.

[34] Xiao W, Chirmule N, Berta SC, McCullough B, Gao G, Wilson JM (1999). Gene therapy vectors based on adeno-associated virus type 1.. J Virol.

[35] Rabinowitz JE, Rolling F, Li C, Conrath H, Xiao W, Xiao X, Samulski RJ (2002). Cross-packaging of a single adeno-associated virus (AAV) type 2 vector genome into multiple AAV serotypes enables transduction with broad specificity.. J Virol.

[36] Halbert CL, Rutledge EA, Allen JM, Russell DW, Miller AD (2000). Repeat transduction in the mouse lung by using adeno-associated virus vectors with different serotypes.. J Virol.

[37] Hildinger M, Auricchio A, Gao G, Wang L, Chirmule N, Wilson JM (2001). Hybrid vectors based on adeno-associated virus serotypes 2 and 5 for muscle-directed gene transfer.. J Virol.

[38] Xiao X, Li J, Samulski RJ (1998). Production of high-titer recombinant adeno-associated virus vectors in the absence of helper adenovirus.. J Virol.

[39] Zolotukhin S, Potter M, Zolotukhin I, Sakai Y, Loiler S, Fraites TJ, Chiodo VA, Phillipsberg T, Muzyczka N, Hauswirth WW, Flotte TR, Byrne BJ, Snyder RO (2002). Production and purification of serotype 1, 2, and 5 recombinant adeno-associated viral vectors.. Methods.

[40] Williams JC, Stone D, Smith-Arica JR, Morris ID, Lowenstein PR, Castro MG (2001). Regulated, adenovirus-mediated delivery of tyrosine hydroxylase suppresses growth of estrogen-induced pituitary prolactinomas.. Mol Ther.

[41] Carrington LM, Southgate T, Saxby LA, Abul-Hassan K, Maleniak TC, Castro MG, Boulton ME (2000). Adenovirus-mediated gene transfer to human lens epithelial cells in organ culture.. J Cataract Refract Surg.

[42] Kabosova A, Kramerov AA, Aoki AM, Murphy G, Zieske JD, Ljubimov AV (2003). Human diabetic corneas preserve wound healing, basement membrane, integrin and MMP-10 differences from normal corneas in organ culture.. Exp Eye Res.

[43] Millar JC, Pang IH, Wang WH, Wang Y, Clark AF (2008). Effect of immunomodulation with anti-CD40L antibody on adenoviral-mediated transgene expression in mouse anterior segment.. Mol Vis.

[44] Klebe S, Sykes PJ, Coster DJ, Krishnan R, Williams KA (2001). Prolongation of sheep corneal allograft survival by ex vivo transfer of the gene encoding interleukin-10.. Transplantation.

[45] Follenzi A, Santambrogio L, Annoni A (2007). Immune responses to lentiviral vectors.. Curr Gene Ther.

[46] Li SD, Huang L (2007). Non-viral is superior to viral gene delivery.. J Control Release.

[47] Borrás T, Gabelt BT, Klintworth GK, Peterson JC, Kaufman PL (2001). Non-invasive observation of repeated adenoviral GFP gene delivery to the anterior segment of the monkey eye in vivo.. J Gene Med.

[48] Liu Y, Okada T, Sheykholeslami K, Shimazaki K, Nomoto T, Muramatsu S, Kanazawa T, Takeuchi K, Ajalli R, Mizukami H, Kume A, Ichimura K, Ozawa K (2005). Specific and efficient transduction of cochlear inner hair cells with recombinant adeno-associated virus type 3 vector.. Mol Ther.

[49] Sharifi BG, Wu K, Wang L, Ong JM, Zhou X, Shah PK (2005). AAV serotype-dependent apolipoprotein A-I_Milano_ gene expression.. Atherosclerosis.

[50] Carlson EC, Liu CY, Yang X, Gregory M, Ksander B, Drazba J, Perez VL (2004). In vivo gene delivery and visualization of corneal stromal cells using an adenoviral vector and keratocyte-specific promoter.. Invest Ophthalmol Vis Sci.

[51] Wentz-Hunter K, Cheng EL, Ueda J, Sugar J, Yue BY (2001). Keratocan expression is increased in the stroma of keratoconus corneas.. Mol Med.

[52] Ku JY, Niederer RL, Patel DV, Sherwin T, McGhee CN (2008). Laser scanning in vivo confocal analysis of keratocyte density in keratoconus.. Ophthalmology.

[53] Ljubimov AV, Huang ZS, Huang GH, Burgeson RE, Gullberg D, Miner JH, Ninomiya Y, Sado Y, Kenney MC (1998). Human corneal epithelial basement membrane and integrin alterations in diabetes and diabetic retinopathy.. J Histochem Cytochem.

